# First karyotype data on the family Myerslopiidae (Hemiptera, Auchenorrhyncha, Cicadomorpha)

**DOI:** 10.3897/CompCytogen.v8i4.8813

**Published:** 2014-11-14

**Authors:** Natalia V. Golub, Valentina G. Kuznetsova, Roman A. Rakitov

**Affiliations:** 1 Zoological Institute, Russian Academy of Sciences, Universitetskaya nab. 1, St. Petersburg 199034, Russia; 2 Paleontological Institute, Russian Academy of Sciences, Profsoyuznaya Ul. 123, Moscow 117997, Russia

**Keywords:** Karyotype, NOR, C-heterochromatin, rDNA, TTAGG telomeric sequence, *Mapuchea chilensis*, Myerslopiidae, Hemiptera, Auchenorrhyncha, Cicadomorpha, Membracoidea

## Abstract

In the first cytogenetic study of the recently proposed family Myerslopiidae the male karyotype of *Mapuchea chilensis* (Nielson, 1996) was analyzed using conventional chromosome staining, AgNOR- and C-bandings, and fluorescence *in situ* hybridization (FISH) with 18S rDNA and (TTAGG)*_n_* telomeric probes. A karyotype of 2n = 16 + XY, NOR on a medium-sized pair of autosomes, subterminal location of C-heterochromatin, and presence of (TTAGG)*_n_* telomeric sequence were determined. Additionally, the male internal reproductive system was studied.

## Introduction

The family Myerslopiidae includes three recent genera of cicadomorphan Auchenorrhyncha with 19 species in New Zealand and temperate Chile (Szwedo 2004). Myerslopiids are small, heavily sclerotized, flightless insects dwelling in leaf litter. The family status of this group, previously classified as a subfamily within Cicadellidae or a tribe within the cicadellid subfamily Ulopinae, was proposed by Hamilton (1999), who argued from morphological evidence that it represents the basal branch of the superfamily Membracoidea (leafhoppers and treehoppers) and shares multiple plesiomorphic characters with Cicadoidea (cicadas), Cercopoidea (froghoppers), or both. This hypothesis received some support from molecular phylogenetic analyses, which recovered myerslopiids outside the rest of Membracoidea (Dietrich et al. 2001, Cryan 2005). Therefore, additional data on these poorly known insects are of considerable interest. We describe here the karyotype of *Mapuchea chilensis* (Nielson, 1996), the data representing the first cytogenetic report on the family Myerslopiidae.

## Material and methods

Four adult males of *Mapuchea chilensis* were collected by the third author in Chile, P.N. Puyehue, Anticura (40.6667°S, 72.1742°W) on 15–17 January 2014 from leaf litter between creeping stems of *Hydrangea serratifolia* (Hooker & Arnott, 1833). Specimens were fixed in 3:1 fixative (96% ethanol: glacial acetic acid) and stored at +4°C. Testes were dissected in a drop of 45% acetic acid and squashed. The cover slip was removed using dry ice. Chromosome staining techniques used were as follows: the Feulgen-Giemsa method (Grozeva and Nokkala 1996) for visualization of standard karyotype; Ag-NOR banding (Howell and Black 1980) for visualization of nucleolus organizing regions, NORs; C-banding (Sumner 1972) for revealing constitutive heterochromatin; and fluorescence *in situ* hybridization (FISH) with 18S rDNA and (TTAGG)*_n_* telomeric probes for detecting the telomeric sequence and the number and chromosomal location of rRNA gene sites (Schwarzacher and Heslop-Harrison 2000). Chromosome slides were analyzed under a Leica DM 6000 B microscope; images were taken with a Leica DFC 345 FX camera using Leica Application Suite 3.7 software with an Image Overlay module.

The classification of cicadomorphan Auchenorrhyncha accepted in this paper follows Dietrich (2005).

## Results

### Reproductive system

**Figure 1.**
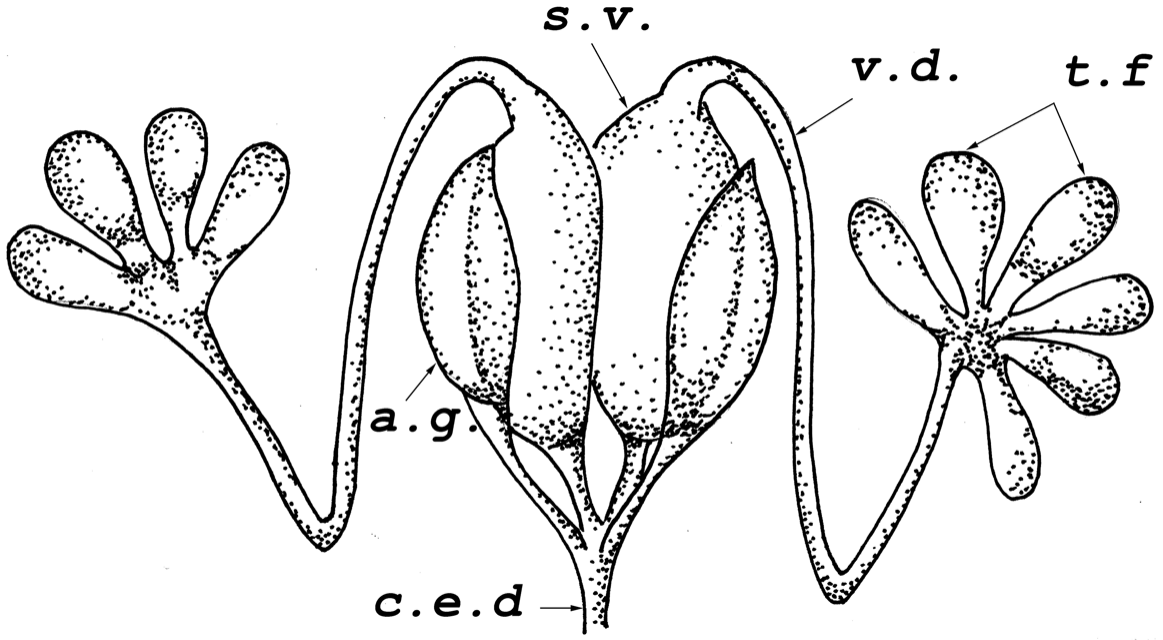
Male reproductive system of *Mapuchea chilensis*. **t.f.** testicular follicles (4 and 6 in different testes); **v.d.** vasa differentia **s.v.** seminal vesicle; **a.g.** accessory gland; **c.d.e.** common ejaculatory duct.

In adult *Mapuchea chilensis* males, the reproductive system consisted of a pair of testes, pair of seminal vesicles, and pair of accessory glands (Fig. 1). In two males, the number of follicles was the same in both testes, 6+6, but in two other males it was 6+5 and 6+4 respectively. The seminal vesicles were cylindrical in shape, fused almost throughout their entire lengths. The accessory glands were oval in shape and narrowed apically.

**Figure 2.**
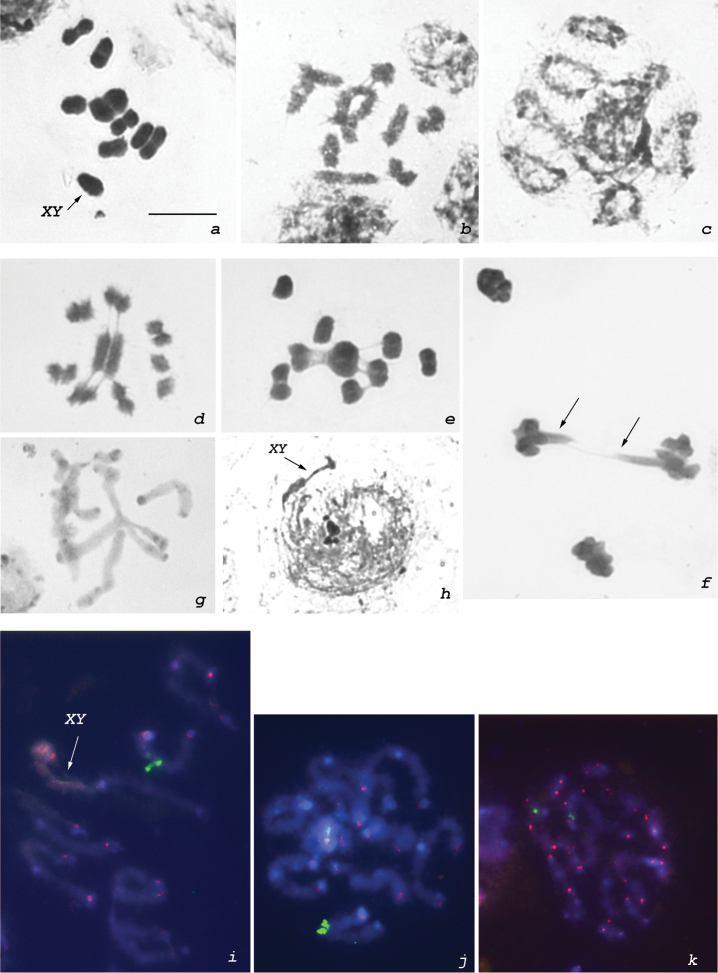
Male karyotype of *Mapuchea chilensis*. **a** metaphase I, n = 8AA + XY **b** diakinesis, largest bivalent with two chiasmata **c** diplotene, 5 bivalents with two chiasmata each **d** metaphase II with the largest chromosome in the center of a ring formed by autosomes. Note chromatin associations between non-homological chromosomes **e** metaphase I showing associations between bivalents **f** anaphase II with lagging chromosomes (arrows) **g** diplotene (C-banding) showing terminal C-bands in chromosomes **h** early prophase (NOR-banding) showing argyrophilic granules associated with autosomes **i–k** diplotenes (**i, j**) and mitotic metaphase (**k**) after FISH with rDNA-probe (green signals) and (TTAGG)*_n_* telomeric probe (red signals). rDNA sites are located on a medium-sized pair of autosomes. Bar = 10µm.

### Standard karyotype

*Mapuchea chilensis* showed a karyotype of 2n = 16 + XY. At MI, 8 bivalents of autosomes and an XY-pair were present (Fig. 2a). One of the bivalents was very large and the others gradually decreased in size. The autosomal bivalents formed one or two subterminal or occasionally interstitial chiasmata (Fig. 2b). In some nuclei, almost all bivalents appeared as rings, evidencing the presence of two subterminal chiasmata (Fig. 2c). At MII, the chromosomes tended to form a ring with the largest bivalent at its center (Fig. 2d). In some cells, non-homological chromosomal associations (Fig. 2d, e) and lagging chromosomes (Fig. 2f) were observed.

### C- and AgNOR-bandings and FISH

After C-banding, the majority of bivalents showed C-blocks at the ends of chromosomes (Fig. 2g). In early prophase cells, a large Ag-positive mass connected with autosomes was identified; in some cases, nucleolar material was present as multiple argyrophilic bodies (Fig. 2h). The 18S rDNA FISH probe localized ribosomal clusters near the ends of one of the medium-sized bivalents (Fig. 2i, j). The (TTAGG)*_n_* telomeric FISH probe produced bright fluorescent signals at the ends of chromosomes (Fig. 2i, j, k).

## Discussion

The number of testicular follicles is generally characteristic of an insect species, although variation between the two testes of the same male has occasionally been reported (Maryańska-Nadachowska et al. 2006, Kuznetsova et al. 2010). The phylogenetic importance of this character in Auchenorrhyncha has been discussed (Emelyanov and Kuznetsova 1983, D’Urso et al. 2005, Kuznetsova et al. 2009, 2010). Despite some intraindividual variation observed in the four examined males, 6 follicles per testis predominated and can thus be considered characteristic of *Mapuchea chilensis*. In Cicadellidae, this number varies from 3 to 14, with low numbers (6 and 4) predominating (Bednarczyk 1993). In other families of Membracoidea, testes with 9 follicles have been recorded in Aetalionidae (Kuznetsova and Kirillova 1993) and testes with 4, 6 and 8 follicles in Membracidae (Emelyanov and Kuznetsova 1983). The number of follicles is higher in other superfamilies of cicadomorphan Auchenorrhyncha: 12-35 in Cercopoidea (Emelyanov and Kuznetsova 1983) and very high (over 100) in Cicadoidea (Glasgow 1908, Moulds 2005).

Among Cicadellidae, chromosome numbers in males vary from 2n = 7(6 + X) to 2n = 27(26 + X) and both X(0) and XY sex chromosome systems occur, the latter being found only occasionally (Kirillova 1988, Wei 2010, Juan 2011). The complement of 2n = 18 (16 + XX/XY), determined for *Mapuchea chilensis*, has been previously described only in two cicadellids, *Taslopa montana* Evans, 1941 from the subfamily Ulopinae (Whitten 1965) and *Hecalus porrectus* (Walker, 1858) from Deltocephalinae (as Thomsoniella (Parabolocratus) albomaculata Distant, 1908 and Thomsoniella (Parabolocratus) porrecta Distant, 1908, see Kirillova 1988). This karyotype has not been recorded so far among Aetalionidae, Membracidae, Cercopoidea, or Cicadoidea (Kirillova 1988, Kuznetsova and Kirillova 1993, Tian and Yuan 1997, Perepelov and Bugrov 2002, Maryańska-Nadachowska et al. 2013).

Therefore, in both the karyotype and the number of follicles, *Mapuchea chilensis* falls within the spectrum of variation observed in Cicadellidae.

Other cytogenetic characters have so far been examined in only a few representatives of cicadomorphan Auchenorrhyncha and thus do not inform on the relationships of Myerslopiidae. *Mapuchea chilensis* was found to have small subterminal C-blocks, the pattern described, with the exception of large blocks in *Philaenus italosignus* Drosopoulos & Remane, 2000 (Cercopoidea: Aphrophoridae) (Maryańska-Nadachowska et al. 2013), in all previously examined species of Cercopoidea (Maryańska-Nadachowska et al. 2013) and Cicadoidea (Perepelov and Bugrov 2002), which are the only other cicadomorphans in which the amount and distribution of C-heterochromatin have been studied. The amount and distribution of C-heterochromatin were found to vary among species of *Philaenus* Stål, 1864 (Maryańska-Nadachowska et al. 2013).

In *Mapuchea chilensis*, rDNA loci were detected by FISH on one of the medium-sized pairs of autosomes, this location being confirmed by AgNOR-staining, which suggested presence of a single autosomal NOR (per haploid set). The latter technique has previously been used to demonstrate variation in the number and position of NORs in four genera of Cercopoidea (Castanhole et al. 2010, Maryańska-Nadachowska et al. 2013); for one of these genera, *Philaenus*, the results have been confirmed using FISH (Maryańska-Nadachowska et al. 2013).

The telomeric sequence (TTAGG)_n_, identified in *Mapuchea chilensis*, is known to be characteristic of the majority of insect groups and is considered to be ancestral for Insecta (Frydrychová et al. 2004, Vitková et al. 2005) and Arthropoda as a whole (Lukhtanov and Kuznetsova 2010). Among Hemiptera, this canonical motif is not present (lost) in the advanced heteropteran infraorders Cimicomorpha and Pentatomomorpha (Grozeva et al. 2011), but has been reported in *Lethocerus patruelis* (Stal, 1854) from the more basal heteropteran infraorder Nepomorpha (Kuznetsova et al. 2012), in coccids (Mohan et al. 2011), aphids (Monti et al. 2011) and the auchenorrhynchan genus *Philaenus* (Maryańska-Nadachowska et al. 2013).
